# *IDH1*-R132 changes vary according to *NPM1* and other mutations status in AML

**DOI:** 10.1038/s41375-018-0299-2

**Published:** 2019-01-08

**Authors:** Brunangelo Falini, Orietta Spinelli, Manja Meggendorfer, Maria Paola Martelli, Barbara Bigerna, Stefano Ascani, Harald Stein, Alessandro Rambaldi, Torsten Haferlach

**Affiliations:** 1 The Institute of Hematology and Research Center for Hemato-Oncological diseases (CREO), University of Perugia, Perugia, Italy; 2 The Institute of Hematology, Ospedale Giovanni XXIII, Bergamo, Italy; 3 The Munich Leukemia Laboratory, Munich, Germany; 4 Pathodiastostik Berlin, Berliner Referenz-und Konsultatios Zentrum fur Lymphoma and Hamatopathologie, Berlin, Germany

**Keywords:** Acute myeloid leukaemia, Cancer genetics

## To the Editor:

Isocitrate dehydrogenase (*IDH1/2*) genes encode for ubiquitinously expressed enzymes that catalyze a redox reaction that converts isocitrate to α-ketoglutarate while reducing NADP to NADPH and liberating CO_2_ [1]. IDH1 exerts his function in the cytoplasm and peroxisomes whilst IDH2 is localized in the mitochondrial matrix [1]. When mutated, the IDH1 and IDH2 enzymes acquire a neomorphic activity leading to the conversion of α-ketoglutarate to D-2-hydroxyglutarate [2–4]. The latter compound acts as an oncometabolite by inhibiting the α-ketoglutarate-dependent enzymes that regulates epigenetic modeling, collagen synthesis and cell signaling [1]. *IDH1* and *IDH2* mutations are mutually exclusive with *TET2* mutations that are known to promote leukemia with a similar mechanism [5].

*IDH1* gene mutations have been detected in 6.6–7.6% [6, 7] of AML patients, most frequently carrying a normal karyotype, and their presence has not been associated with prognostic relevance. They are heterozygous missense mutations confined to a single arginine residue, R132, in the enzyme active site [1]. Five R132 mutations leading to different amino acid exchanges have been described [6, 7]: p.R132H, p.R132C, p.R132G, p.R132S, and p.R132L, with R132H being the most frequent [7]. As a whole group, the *IDH1-*R132 mutations are more frequent in cases carrying *NPM1* mutations [6, 7] but it is yet unknown how the amino acid substitution of arginine at position 132 correlates with the mutational status of *NPM1* and other mutations in AML. Here, combining molecular analyses and immunohistochemistry we demonstrate that the R132H and R132C substitutions show a different distribution pattern among AML genotypes.

We first investigated 140 AML patients with normal cytogenetics enrolled in Northern Italy Leukemia Group (NILG) multicenter clinical trial (NCT00495287), for which both molecular and immunohistochemical data were available (Supplementary Information). In all 140 patients, the results of next generation sequencing (NGS) for *IDH1* and *NPM1* mutations were blindly compared with those of immunohistochemistry on bone marrow (BM) biopsies using monoclonal antibodies against IDH1-R132H and NPM1, respectively. The antibody against the IDH1-R132H mutant was previously produced by Capper et al. [8] and extensively investigated in various kind of tumors. The antibody directed against the nucleophosmin (NPM1) [9] was generated in BF laboratory. Cytoplasmic nucleophosmin-1 expression was regarded as predictive of *NPM1* mutations [9, 10] (Supplementary Information). For all studies described below, written informed consent to examine leukemic samples was obtained in accordance with the Declaration of Helsinki and approval was obtained from Local Ethic Committee.

**Fig. 1:**
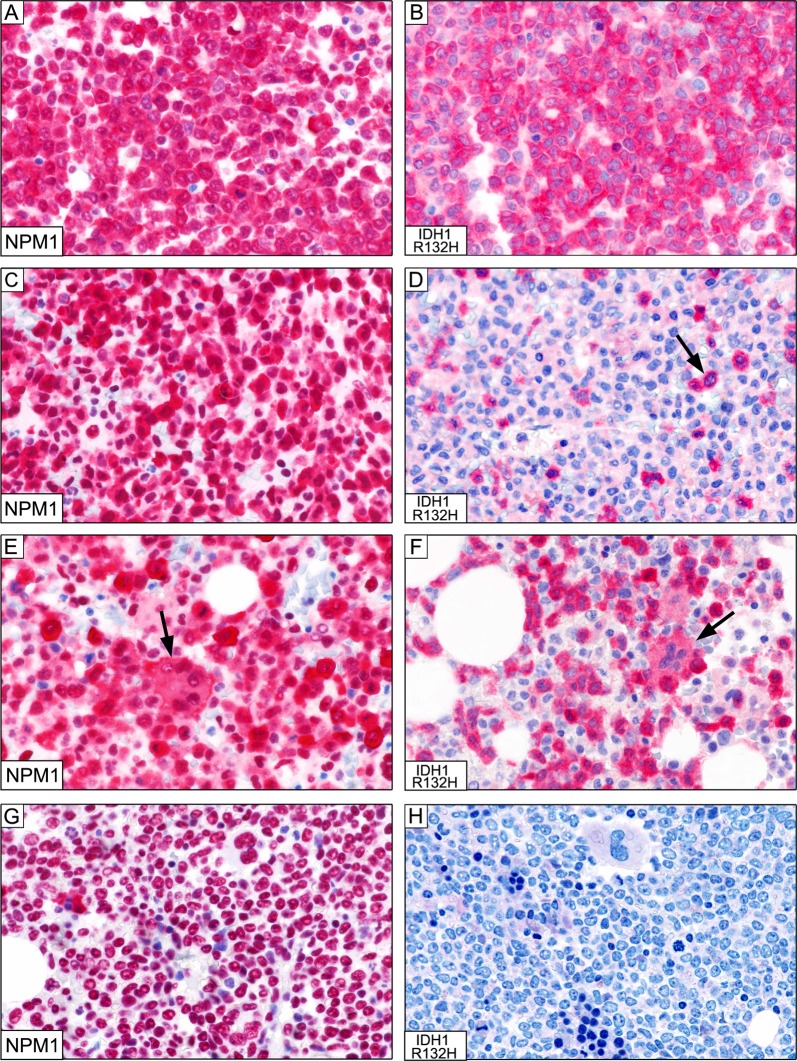
**a** Massive infiltration of BM by *NPM1*-mutated AML cells showing the expected nuclear plus aberrant cytoplasmic positivity for nucleophosmin-1 (×400). **b** The same case as (**a**), showing a comparable number of leukemic cells expressing the IDH1-R132H mutant; positivity is mostly restricted to the cytoplasm of blast cells (×400). **c** Marked infiltration of BM by *NPM1*-mutated AML cells showing the expected nuclear plus aberrant cytoplasmic positivity for nucleophosmin-1. The rare elements with nucleus-restricted positivity for NPM1 represent residual normal hematopoietic cells (×400). **d** The same case as (**c**), showing that leukemic cells expressing the IDH1-R132H mutant represent only a small subclone of the total population of *NPM1*-mutated cells (×400). **e** Marked infiltration of BM by *NPM1*-mutated AML cells showing the expected nuclear plus aberrant cytoplasmic positivity for nucleophosmin-1 (×400). The arrow points to a positive megakaryocyte. Elements with nucleus-restricted positivity for NPM1 represent normal residual hematopoietic cells (×400). **f** The same case as (**c**), showing that the percentage of leukemic cells expressing the IDH1-R132H is slightly inferior to that of NPM1 cytoplasmic-positive cells. As in (**e**), the IDH1-R132H mutant is present both in mononuclear blast cells and in a megakaryocyte (arrow). The IDH1-R132H negative cells represent normal residual hematopoietic cells (×400). **g** Massive bone marrow infiltration by leukemic cells with nucleus-restricted positivity for nucleophosmin-1 (predictive of absence of *NPM1* mutations, confirmed molecularly) (×400). **h** Specificity of the antibody against IDH1-R132H is demonstrated by the negativity of leukemic cells molecularly carrying the *IDH1*-R132C mutation (×400). (**a**–**h**) Dako REAL Detection System Alkaline Phosphatase/RED rabbit/mouse

Molecular analyses revealed *NPM1* mutations in 71/140 (51%) cases. These findings were fully confirmed by immunohistochemistry that showed cytoplasmic NPM1 (predictive of *NPM1* mutations) (Fig. 1a, c, e) in the same 71 cases. In the remaining 69 cases, NPM1 expression was nucleus-restricted, as expected in cases with *NPM1* wild-type status [9].

Molecular analyses revealed *IDH1*-R132H mutations in 10/140 (7%) cases. Notably, these 10 cases were all *NPM1*-mutated and showed cytoplasmic NPM1 at immunohistochemistry (10/71:14%). The same 10 cases, revealed R132H mutant expression at cytoplasmic level (Fig. 1b, d, f), as expected for the cytosolic function of the enzyme [1]. At diagnosis, the percentage of IDH1-R132H-positive leukemic cells and with aberrant cytoplasmic NPM1 were comparable in 6/10 cases (representative examples are shown in Fig. 1a, b), whilst in 4/10 cases the IDH1-R132H-positive leukemic cells accounted for only a fraction of them, ranging between 3% and 70%, strongly suggesting that they represented a subclone. A representative example showing about 5–10% of IDH1-132H-positive leukemic cells in shown in Fig. 1d.

Extended molecular analysis of the 140 cases also detected *IDH1* mutations other than p.R132H in 8/140 (6%) cases. In particular: p.R132C in 3/140 cases (2%; 2 *NPM1*-mutated, 1 *NPM1-*wt), p.R132G in 2/140 cases (1%; both *NPM1*-mutated), and p.R132S in 3/140 cases (2%; all *NPM1*-mutated). Notably, all these eight cases were negative with the mAb specific for IDH1-R132H (Fig. 1g, h).

**Fig. 2:**
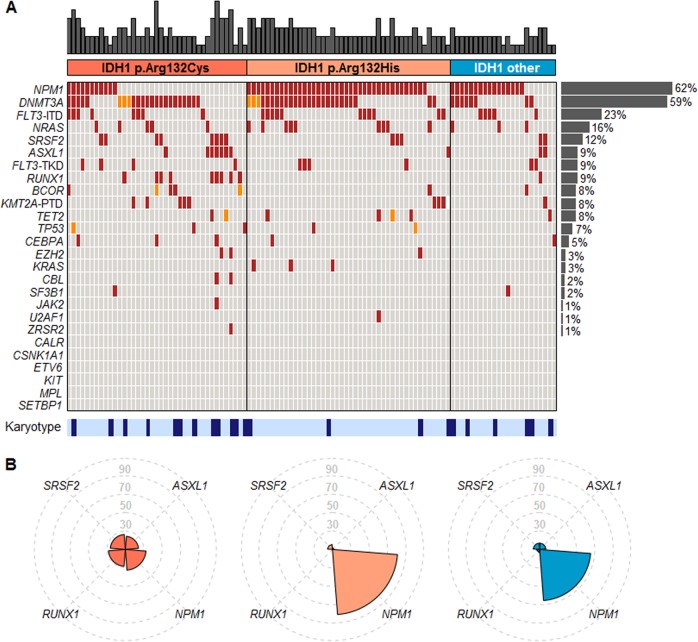
**a** Molecular and cytogenetic characterization of *IDH1*-mutated patients. Illustration of all 106 samples, each column represents one patient. All 25 additionally analyzed genes as well as karyotype information are given for each patient. Patients are grouped by *IDH1* R132C, R132H, and R132 other. Light gray: wild type, red: mutated, orange: variant of uncertain significance, dark blue: aberrant karyotype, light blue: normal karyotype, white: no data available. The number of additional mutations per patient is illustrated as bar chart above the graph. The mutation frequencies of single genes are given as bar chart at the right. **b** Spider plot illustrating the mutation frequencies (in %) of *ASXL1*, *NPM1*, *RUNX1*, and *SRSF2* mutations for the single groups of *IDH1* R132C, R132H, and R132 other

To further validate the above findings and extend the correlation of *IDH1*-R132 changes to other mutations, we analyzed at Munich Leukemia Laboratory another independent cohort of *IDH1*-mutated AML by comprehensive gene sequencing. Our previously described AML cohort [11] comprised 106 *IDH1*-mutated de novo AML patients, most often showing *IDH1*-R132H (*n* = 44/106; 41%) and R132C (39/106; 37%). In this study, we investigated all cases by NGS and gene scan targeting *IDH1* and *NPM1* beside 25 other genes (Supplemental Information). 62% (66/106) cases of *IDH1*-mutated patients showed also a *NPM1* mutation, 48% a *DNMT3A* mutation, 23% a *FLT3*-ITD, 16% a *NRAS* mutation, and 12% a *SRSF2* mutation (Fig. 2a; Supplementary Table 1). All other mutations occurred in <10% of cases. Therefore, we could confirm the high association of *IDH1*-R132H with *NPM1* mutations in this cohort. In fact, 39/44 (89%) *IDH1*-R132H patients showed a *NPM1* mutation, while in only 44% (27/62) of the other *IDH1*-R132 mutated patients a *NPM1* mutation occurred (*p* < 0.001) (Fig. 2a; Supplementary Table 1).

Analysis of further gene mutations and their associations showed that *IDH1-*R132H was mutually exclusive for *RUNX1* (0/44; 0%; *p* = 0.001), *SRSF2* (3/44; 7%; *p* = 0.104) and *ASXL1* (1/44; 2%; *p* = 0.02 3) and were less frequently mutated compared to *IDH1-*R132C mutated patients (23%, 21%, and 18%, respectively) (Fig. 2a, supplementary Table 1). These data resulted, therefore, in two different mutation patterns, differentiating *IDH1-*R132H and R132C mutated AML (Fig. 2b). While R132C shows a more s-AML like genetic, R132H shows a typical de novo AML pattern [12]. The third group of *IDH1*-mutated patients (other than R132H/C) seemed to be a mixture of both patterns (Fig. 2b). Addressing the prognostic impact of these *IDH1-*R132 variants showed a slightly worse prognostic impact of *IDH1-*R132C compared to *IDH1-*R132H-mutated patients (overall survival: 19.9 versus 24.9 months; Supplementary Figure 1).

Different co-mutation patterns for hotspots within genes has been previously described under various circumstances [13]. As an example, the *NPM1* mutation preferentially associates with *NRAS-*G12/13 but not with *NRAS-*Q61 [13]. These findings strongly suggest that the functional consequences of hotspot mutations within genes may not be equivalent. At present, no compound *NPM1*-mutated/*IDH1*-mutated mouse model has been described.

Is there any utility to have an anti-IDH1-R132-specific antibody in the NGS era? Although, molecular analyses remain the gold standard for the identification of *IDH1* mutations, immunohistochemistry may be a useful adjunct to the above techniques, particularly in hematological centers that still use BM biopsies. Under these circumstances, the antibody could be used both at diagnosis and for monitoring of AML after chemotherapy or targeted therapy with IDH1 inhibitors [14]. The antibody would also allow to analyze the genetic lesion at protein level in the tissues and provide information related to the topographical distribution (nearby trabeculae or vessels) of leukemic cells. Moreover, the use of the antibody may be particularly important in cases of “punctio sicca” or myeloid sarcoma, especially when scarce material is available for molecular analyses (e.g. punch biopsies of the skin).

## Supplementary information

Supplemental material
