# Supplementary material for: IDH1-R132 changes vary according to NPM1 and other mutations status in AML
Source: Leukemia. 2019 Jan 8;33(4):1043–7. doi: 10.1038/s41375-018-0299-2 (PMC6484707; doi:10.1038/s41375-018-0299-2)
Supplement: Supplementary file 1 — Supplemental material [file 41375_2018_299_MOESM1_ESM.docx]

**SUPPLEMENTARY INFORMATION**

**1. Immunohistochemistry and molecular analysis in 140 patients with normal cytogenetics AML from the Northern Italian Leukemia Group (NILG)**

**1.1 Immunohistochemical procedures with antibodies against NPM1 and IDH1 R132H**

All bone marrow (BM) biopsies were centralized at the Institute of Hematology, University of Perugia, Italy, fixed in B5 for 2 hours, decalcified in EDTA for 5 hours, dehydrated and then processed for paraffin embedding. Paraffin sections from each BM biopsy were immunostained with a mouse mAb anti-NPM1 (generated in BF laboratory, recognizing both the wild-type and mutated NPM1 protein) and defined to carry an *NPM1* mutation, if leukemic cells showed aberrant cytoplasmic expression of nucleophosmin. Paraffin sections were immunostained in parallel with a highly specific mAb direct against the IDH1-R132H mutant (clone H09).

Before immunostaining, BM paraffin sections were subjected to antigen retrieval. For both antibodies, unmasking was performed by incubating the paraffin sections in buffer EDTA pH 9.0 (EnVision FLEX Target Retrieval Solution High pH (50x) Dako-Agilent (code K8004). Unmasking for NPM1 was at 85C°, 5 minutes; unmasking for IDH1 R132H was at 97C°, 10 minutes.

Primary antibodies were diluted in Dako buffer (code S2022) (anti-NPM1 clone 376 at 1:5 dilution and anti-IDH1 R132H mAb, clone H09, at 1:40 dilution) and incubated with paraffin sections for 30 minutes.

Amplification with Mouse linker was only employed with the anti-IDH1 R132H mAb.

All sections were processed in a Dako Autostainer using the Dako REAL Detection System Alkaline Phosphatase/RED rabbit/mouse (code K5005).

Sections were then counterstained in hematoxylin for 5 minutes and mounted in Kaiser’s glycerol gelatin (Merck KGA, Germany; code 1.09242.1100).

**1.2 Molecular Studies: comparison with immunohistochemical data in the NILG clinical trial**

*NPM1* mutations were prospectively evaluated within the clinical protocol NILG 02/06 on genomic DNA by PCR amplification and Sanger sequencing as previously described (Falini B et al., NEJM, 2005, 254-262).

*IDH1* mutations identification was obtained using Next Generation Sequencing (NGS) commercial kits (Trusight Myeloid panel (Illumina, San Diego, CA) or Sophia Myeloid Solution (SOPHiA GENETICS, SA, CH) on an Illumina Platform (MiSeq or MiniSeq). The somatic variations were defined using public databases. The mean coverage was 5600 reads for IDH1 region. The detection limit for single nucleotide variants was set to 5% variant allele frequency. IDH1 mutations below 5% were manually checked. Samples with IDH1 VAF >20% were confirmed with PCR (primers: IDH1F-TGTGTTGAGATGGACGCCTA, IDH1R-TGCCATCACTGCAGTTGTAG, annealing temperature: 58°C) and Sanger sequencing.

**2. Patients and methods (validation cohort: Munich Leukemia Laboratory)**

***2.1 Patient cohorts***

In a starting cohort of 1,394 patients diagnosed with *de novo* AML 106 patients with *IDH1* mutation were identified (7.6%). Follow-up data was available for 96/106 *IDH1* mutated patients. For overall survival analyses a *IDH1* wild type FAB and MRC matched reference cohort was randomly assembled to minimize bias based on diverging risk classifications (n=540). All cases were intensively treated with standard chemotherapy.^1-3^ The median follow-up was 53 months for the *IDH1* mutant cohort and 58 months for the FAB/MRC matched *IDH1* wild type reference cohort. All cases were included in a previous study.^4^

Samples were referred to the MLL Munich Leukemia Laboratory between October 2005 and January 2017 for diagnostic assessment. Diagnosis was performed on bone marrow smears according to standard World Health Organization (WHO) and FAB criteria.^5^ Cytogenetics was available in all cases.

All patients gave their written informed consent for scientific evaluations. The study design adhered to the tenets of the Declaration of Helsinki and was approved by our institutional review board before its initiation.

***2.2 Next generation sequencing***

All 106 *IDH1* mutated patients were analyzed by a myeloid gene panel containing *ASXL1*, *BCOR*, *CALR*, *CBL*, *CEBPA*, *CSNK1A1*, *DNMT3A*, *ETV6*, *EZH2*, *FLT3*-TKD, *IDH1*, *IDH2*, *JAK2*, *KIT*, *KRAS*, *MPL*, *NPM1*, *NRAS*, *RUNX1*, *SETBP1*, *SF3B1*, *SRSF2*, *TET2*, *TP53, U2AF1* and *ZRSR2*. The library of 26 genes was generated with a TruSeq Custom Amplicon Low Input Kit (Illumina, San Diego, CA), following the manufacturers’ protocol. The library was sequenced and demultiplexed on a MiSeq instrument (Illumina, San Diego, CA) as described previously.^6^ The FASTQ files were further processed using the Sequence Pilot software version 4.1.1 Build 510 (JSI Medical Systems, Ettenheim, Germany) for alignment and variant calling. Analysis parameters were set according to manufacturers’ default recommendation. Validity of the somatic mutations was checked against the publicly accessible COSMIC v69 database (http://cancer.sanger.ac.uk/cancergenome/projects/ cosmic) and functional interpretation was performed using SIFT 1.03 (http://sift.jcvi.org), PolyPhen 2.0 (http://genetics.bwh.harvard.edu/pph2) and MutationTaster 1.0 algorithms (http://www.mutationtaster.org).^7^ Additionally, *TP53* variants were verified using the IARC repository.^8^ Single nucleotide polymorphisms (SNP) were annotated according to the NCBI dbSNP (http://www.ncbi.nlm.nih.gov/snp; Build 137) database. The detection limit/sensitivity for single nucleotide variants was 3% variant allele frequency, for GC-rich sequences the detection limit was set to 5% variant allele frequency (e.g. *CEBPA* or homopolymeric region in *ASXL1*). Variants of uncertain significance were excluded from statistical analyses.

***2.3 Gene scan and quantitative PCR***

*MLL*-PTD was analyzed with a quantitative PCR assay, *FLT3*-ITD was analyzed by gene scan, both described methodically previously.^9, 10^ Detection limit for *FLT3*-ITD was 5%.

***2.4 Statistical analyses***

Dichotomous variables were compared between different groups using the χ^2^-test. Results were considered significant at *p*<0.05. Adjustment for multiple testing was not done. Statistical analyses were performed using SPSS version 19.0 (IBM Corporation, Armonk, NY); the reported *p*-values are two-sided. Survival curves were calculated for overall survival (OS) according to Kaplan-Meier and compared using the two-sided log rank test. OS was defined as the time from diagnosis to last follow-up or death.

**3. Results (validation cohort: Munich Leukemia Laboratory)**

***3.1 Mutational characterization of IDH1 mutated patients***

106 *IDH1* mutated patients were identified in the *de novo* AML cohort. Addressing the amino acid exchange at position R132 revealed that R132H was the most frequently occurring exchange (n=44), followed by R132C (n=39), R132G (n=11), R132S and R132L (both n=6). Therefore, the three less frequently appearing patient groups were grouped to R132 other. The co-occuring mutations in genes other than *IDH1* as well as the karyotype information are given in Supplementary Table 1.

**Supplementary Table 1: Mutational characterization of the patient groups.**

|  | ***IDH1***  **(n=106)** | ***IDH1* R132C**  **(n=39, 37%)** | ***IDH1* R132H**  **(n=44, 41%)** | ***IDH1* R132 other**  **(n=23, 22%)** |
| --- | --- | --- | --- | --- |
| **Gene mutation, n (%)** | | | | |
| ***ASXL1*** | **10 (9%)** | **7 (18%)** | **1 (2%)** | **2 (9%)** |
| *BCOR* | 5 (5%) | 3 (8%) | 1 (2%) | 1 (4%) |
| *CALR* | 0 (0%) | 0 (0%) | 0 (0%) | 0 (0%) |
| *CBL* | 2 (2%) | 2 (5%) | 0 (0%) | 0 (0%) |
| *CEBPA* | 5 (5%) | 3 (8%) | 1 (2%) | 1 (4%) |
| *CSNK1A1* | 0 (0%) | 0 (0%) | 0 (0%) | 0 (0%) |
| *DNMT3A* | 51 (48%) | 20 (51%) | 23 (52%) | 8 (35%) |
| *ETV6* | 0 (0%) | 0 (0%) | 0 (0%) | 0 (0%) |
| *EZH2* | 3 (3%) | 2 (5%) | 1 (2%) | 0 (0%) |
| *FLT3*-ITD | 24 (23%) | 8 (21%) | 11 (25%) | 5 (22%) |
| *FLT3*-TKD | 10 (9%) | 4 (10%) | 4 (9%) | 2 (9%) |
| *IDH2* | 3 (3%) | 1 (3%) | 2 (5%) | 0 (0%) |
| *JAK2* | 1 (1%) | 1 (3%) | 0 (0%) | 0 (0%) |
| *KIT* | 0 (0%) | 0 (0%) | 0 (0%) | 0 (0%) |
| *KMT2A-*PTD | 9 (8%) | 5 (13%) | 3 (7%) | 1 (4%) |
| *KRAS* | 3 (3%) | 0 (0%) | 3 (7%) | 0 (0%) |
| *MPL* | 0 (0%) | 0 (0%) | 0 (0%) | 0 (0%) |
| ***NPM1*** | **66 (62%)** | **11 (28%)** | **39 (89%)** | **16 (70%)** |
| *NRAS* | 17 (16%) | 5 (13%) | 9 (20%) | 3 (13%) |
| ***RUNX1*** | **10 (9%)** | **9 (23%)** | **0 (0%)** | **1 (4%)** |
| *SETBP1* | 0 (0%) | 0 (0%) | 0 (0%) | 0 (0%) |
| *SF3B1* | 2 (2%) | 1 (3%) | 0 (0%) | 1 (4%) |
| ***SRSF2*** | **13 (12%)** | **8 (21%)** | **3 (7%)** | **2 (9%)** |
| *TET2* | 5 (5%) | 1 (3%) | 3 (7%) | 1 (4%) |
| *TP53* | 3 (5%) | 2 (5%) | 1 (2%) | 0 (0%) |
| *U2AF1* | 1 (1%) | 0 (0%) | 1 (2%) | 0 (0%) |
| *ZRSR2* | 1 (1%) | 1 (3%) | 0 (0%) | 0 (0%) |
| **Karyotype, n (%)** | | | | |
| Normal | 84 (79%) | 27 (69%) | 40 (91%) | 17 (74%) |
| aberrant | 22 (21%) | 12 (31%) | 4 (9%) | 6 (26%) |

***3.2 Prognostic information***

The prognostic influence on overall survival for *IDH1* R132C, R132H, R132 other and *IDH1* R132 wild type cases is shown as Kaplan-Meier plots in Supplementary Figure 1.


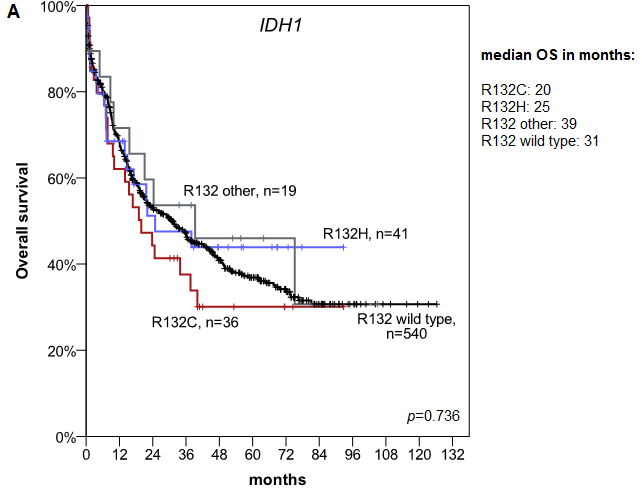


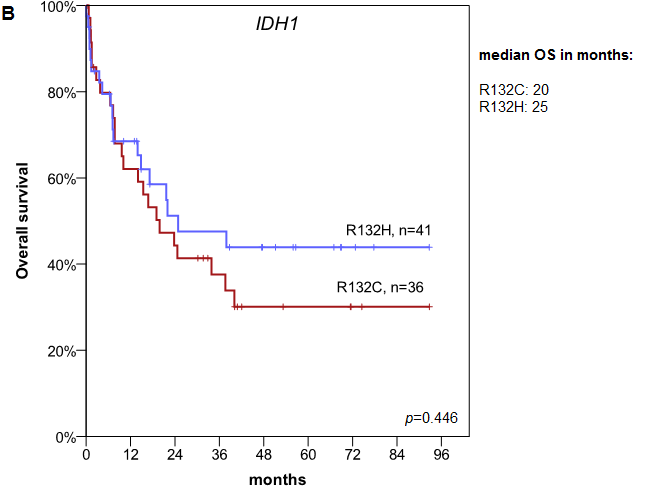


**Supplementary Figure 1**. A) Overall survival according to *IDH1* R132C, R132H, R132 other and R132 wild type. B) Overall survival according to *IDH1* R132C and R132H. Case numbers (n), median overall survival and *p*-values are given. OS: overall survival.

**References**

1 Büchner T, Schlenk RF, Schaich M, Dohner K, Krahl R, Krauter J, et al. Acute Myeloid Leukemia (AML): Different Treatment Strategies Versus a Common Standard Arm--Combined Prospective Analysis by the German AML Intergroup. *J Clin Oncol* 2012; **30**: 3604-3610.

2 Bennett JM, Catovsky D, Daniel MT, Flandrin G, Galton DA, Gralnick HR, et al. Proposals for the classification of the acute leukaemias. French-American-British (FAB) co-operative group. *Br J Haematol* 1976; **33**: 451-458.

3 Grimwade D, Hills RK, Moorman AV, Walker H, Chatters S, Goldstone AH, et al. Refinement of cytogenetic classification in acute myeloid leukemia: determination of prognostic significance of rare recurring chromosomal abnormalities among 5876 younger adult patients treated in the United Kingdom Medical Research Council trials. *Blood* 2010; **116**: 354-365.

4 Meggendorfer M, Cappelli LV, Walter W, Haferlach C, Kern W, Falini B, et al. IDH1R132, IDH2R140 and IDH2R172 in AML: different genetic landscapes correlate with outcome and may influence targeted treatment strategies. *Leukemia* 2018.

5 Arber DA, Orazi A, Hasserjian R, Thiele J, Borowitz MJ, Le Beau MM, et al. The 2016 revision to the World Health Organization (WHO) classification of myeloid neoplasms and acute leukemia. *Blood* 2016; **127**: 2391-2405.

6 Delic S, Rose D, Kern W, Nadarajah N, Haferlach C, Haferlach T, et al. Application of an NGS-based 28-gene panel in myeloproliferative neoplasms reveals distinct mutation patterns in essential thrombocythaemia, primary myelofibrosis and polycythaemia vera. *Br J Haematol* 2016.

7 Schwarz JM, Rodelsperger C, Schuelke M, Seelow D. MutationTaster evaluates disease-causing potential of sequence alterations. *Nat Methods* 2010; **7**: 575-576.

8 Petitjean A, Mathe E, Kato S, Ishioka C, Tavtigian SV, Hainaut P, et al. Impact of mutant p53 functional properties on TP53 mutation patterns and tumor phenotype: lessons from recent developments in the IARC TP53 database. *Hum Mutat* 2007; **28**: 622-629.

9 Schnittger S, Kinkelin U, Schoch C, Heinecke A, Haase D, Haferlach T, et al. Screening for MLL tandem duplication in 387 unselected patients with AML identify a prognostically unfavorable subset of AML. *Leukemia* 2000; **14**: 796-804.

10 Schnittger S, Schoch C, Kern W, Staib P, Wuchter C, Sauerland MC, et al. FLT3 length mutations in AML: Correlation to cytogenetics, FAB- subtype, and prognosis in 652 patients. *Blood* 2000; **96**: 826a.
